# Cardiac Rehabilitation: A Bibliometric Review From 2001 to 2020

**DOI:** 10.3389/fcvm.2021.672913

**Published:** 2021-05-31

**Authors:** Guozhen Yuan, Jingjing Shi, Qiulei Jia, Shuqing Shi, Xueping Zhu, Yan Zhou, Shuai Shi, Yuanhui Hu

**Affiliations:** ^1^Department of Cardiovascular Diseases, Guang'anmen Hospital, China Academy of Chinese Medical Sciences, Beijing, China; ^2^Department of General Internal Medicine, Guang'anmen Hospital, China Academy of Chinese Medical Sciences, Beijing, China

**Keywords:** cardiac rehabilitation, cardiovascular disorders, COVID-19, mapping knowledge domains, CiteSpace

## Abstract

Cardiovascular disease (CVD) is a serious threat to global public health due to its high prevalence and disability rate. Meanwhile, cardiac rehabilitation (CR) has attracted increasing attention for its positive effects on the cardiovascular system. There is overwhelming evidence that CR for patients with CVD is effective in reducing cardiovascular morbidity and mortality. To learn more about the development of CR, 5,567 papers about CR and related research were retrieved in the Web of Science Core Collection from 2001 to 2020. Then, these publications were scientometrically analyzed based on CiteSpace in terms of spatiotemporal distribution, author distribution, subject categories, topic distribution, and references. The results can be elaborated from three aspects. Firstly, the number of annual publications related to CR has increased year by year in general over the past two decades. Secondly, a co-occurrence analysis of the output countries and authors shows that a few developed countries such as the United States, Canada, and the UK are the most active in carrying out CR and where regional academic communities represented by Sherry Grace and Ross Arena were formed. Thirdly, an analysis of the subject categories and topic distribution of the papers reveals that CR is a typical interdiscipline with a wide range of disciplines involved, including clinical medicine, basic medicine, public health management, and sports science. The research topics cover the participants and implementers, components, and the objectives and requirements of CR. The current research hotspots are the three core modalities of CR, namely patient education, exercise training and mental support, as well as mobile health (mHealth) dependent on computer science. In conclusion, this work has provided some useful information for acquiring knowledge about CR, including identifying potential collaborators for researchers interested in CR, and discovering research trends and hot topics in CR, which can offer some guidance for more extensive and in-depth CR-related studies in the future.

## Introduction

Cardiovascular disease (CVD) is prevalent and is causing deaths worldwide, whether in developed or developing countries. It was reported that about 485.6 million people suffered from CVD, and 17.8 million deaths were attributed to it in 2017 globally, an increase of 21.1 and 28.5% compared with a decade ago, respectively (1). What is worse is that CVD is expected to account for >22.2 million deaths by 2030 (2). In the United States, the overall prevalence of CVD (comprising coronary heart disease, heart failure, stroke, and hypertension) in adults ≥20 years of age is 48.0% in 2013 and 2016 (3). In China, according to the latest estimate of the National Center for Cardiovascular Diseases, there are 330 million CVD cases, resulting in 40% of the deaths in the nation (4). On the other hand, advances in medical technology and a younger age of onset have made a sharp increase in cases surviving with CVD. All in all, non-communicable diseases represented by CVDs, especially ischemic heart disease, have become the leading cause of the growing global disease burden in the past 30 years (5). Therefore, preventing the occurrence and recurrence of CVD is a great challenge for the public health systems worldwide. Hopefully, mounting scientific evidences show that cardiac rehabilitation (CR) plays a positive role in the secondary prevention of CVD, including lowering cardiovascular risk and mortality, reducing relapse and hospital admissions, and improving cardiopulmonary functions, quality of life, and prognosis (6–12). CR has been an indispensable part of modern cardiology, endorsed and recommended by some authoritative organizations, such as the American Heart Association (AHA), American College of Cardiology, and the European Society of Cardiology (13, 14).

Actually, the development of CR is out of balance in different regions and countries. According to a survey of global CR availability and density, CR is available in only half of the countries around the world, and the capacity is grossly insufficient, where offered (15). Although it is difficult to conduct a systematic epidemiological investigation on the current situation of CR around the world, it does not mean we cannot maximize knowledge gain about CR, nevertheless. Visual analytics of the literature offers a valuable, timely, repeatable, and flexible approach besides traditional systematic reviews so as to track the new emerging trends and identify critical evidence (16). In this review, visual analysis based on CiteSpace is applied in published literatures on the topic of CR in the last 20 years to help learn the trends of this old and young discipline in the new century and to promote extensive application in CVD patients.

## Materials and Methods

### Data Source and Search

The publications were obtained from the Core Collection database of Web of Science (WoS) (http://apps.webofknowledge.com) because it is considered the most prominent database of scientific publications on many research topics. The strategy used during the search was [TS = (“cardiac rehabilitation” OR “heart rehabilitation”)] AND [Language = (English)]. A total of 7,678 results were found from 2001 to 2020 (retrieved on November 19, 2020). Then, the exclusion of publications was performed according to document type; therefore, only 5,567 records (4,721 articles and 846 reviews) were used in the final database. All the records, including the titles, authors, abstracts, keywords, cited references, and so on, were then imported to CiteSpace5.7. R2, a knowledge mapping tool based on Java to visualize the patterns and trends in scientific literature (17, 18).

### Scientometric Analysis Methods

CiteSpace, a freely available Java-based application, was designed to analyze and visualize trends and patterns in scientific literature, presenting the structure and distribution of scientific knowledge. It focuses on finding critical points in the development of a field or a domain, especially intellectual turning points and pivotal points (17). Once the literature has been imported into CiteSpace, the first step is data cleaning. If there are no duplicates, the original data can be used directly; otherwise, the repeat ones should be removed before subsequent analysis. CiteSpace supports structural and temporal analyses of a range of networks derived from scientific publications, including collaboration networks, co-occurrence networks, and co-citation networks, and it also supports networks of hybrid node types such as terms, authors, and countries. The results are displayed in the form of visual graphs where nodes represent research items; the more frequently the item appears or is cited, the larger is the size of the node. Links between nodes describe a co-occurrence or a co-citation between these nodes, and their thickness indicates the strength of these correlations: the thicker the line, the closer is the connection between them. The shade or tone of the node and link color indicates the chronological order of occurrence of the item (16). Burst detection is also one of the features of CiteSpace, and a positive node after burst detection means a sharp change in its frequency in a short period of time (19). Such nodes usually suggest a shift in a certain field of research and are shown in red in the knowledge map. In addition, CiteSpace also introduces some common network topology parameters to describe the structure of complex networks, such as betweenness centrality (BC) put forward by the American sociologist Linton Freeman. The BC of node X represents the ratio of the shortest paths between any two different nodes through node X in all the shortest paths of these two nodes in a fully connected network (20). At the documental level, the betweenness centrality metric can be used to partially assess the importance of each node in the network (21). A node with BC >0.1 is displayed in terms of a purple ring. The thickness of the purple ring increases as the degree of its BC rises, which is a measure associated with the transformative potential of a scientific contribution (16). What is more is that cluster analysis is another important way of analyzing knowledge networks easily in CiteSpace. More specifically, terms in the literature are classified based on their similarity and are scored by some specific algorithms, and then the term with the highest score of each cluster is selected as the representative, that is, the label of the cluster. The size of the cluster is the number of grouped objects. CiteSpace distributes the ID #0 to the largest cluster formed, the ID #1 to the second largest, and so on (22). It should be noted that all running parameters were set as defaults for the following analysis, except for the time slice setting of 2 years.

## Results and Discussion

### Annual Quantitative Distribution of Publications

**Figure 1:**
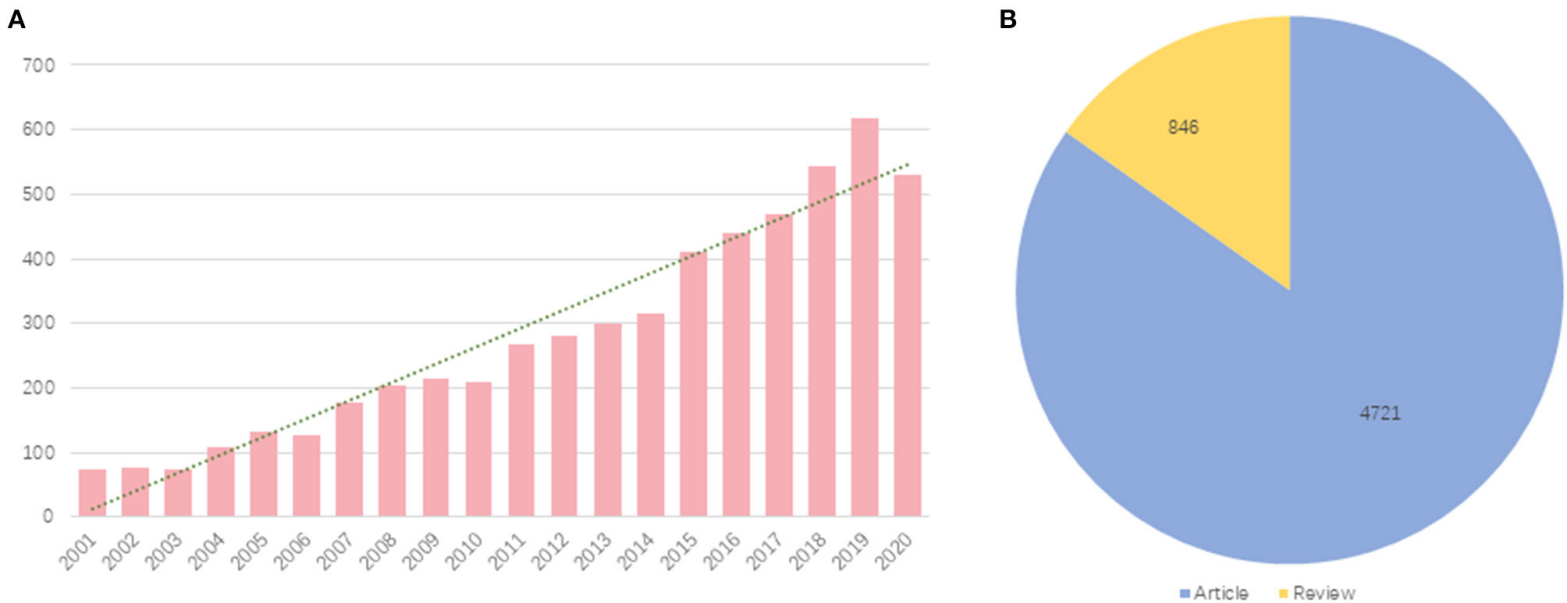
Yearly quantity and document type of publications about cardiac rehabilitation (CR) and related research. **(A)** Annual quantitative distribution. **(B)** Document type. *Blue* represents articles and *yellow* represents reviews.

The annual number of published papers reflects the pace of subject knowledge and is a significant indicator for studying the trends in the field (23). The yearly quantity and the document type of publications of research on CR are visually displayed in Figure 1. We can draw directly that the number of CR-related literature has increased year by year on the whole between 2001 and 2020, except for a slight decrease in 2006 and 2010 (Figure 1A), indicating a steady development of CR. Especially since the last 5 years, CR has stepped into a period of rapid development, with the quantity of papers growing much faster than that in the previous decade, which shows that CR is getting more and more attention. It may be attributed to the worldwide increase in CVD patients on the one hand and, on the other hand, perhaps implies that the concept of CR is popularizing from local to global. Furthermore, articles account for about 85% in terms of document type (Figure 1B), which indicates the greater emphasis paid on clinical practice and that original studies such as case report and clinical trials are the mainstream in the area of CR.

### Country Ranking and Co-author Analysis

**Figure 2:**
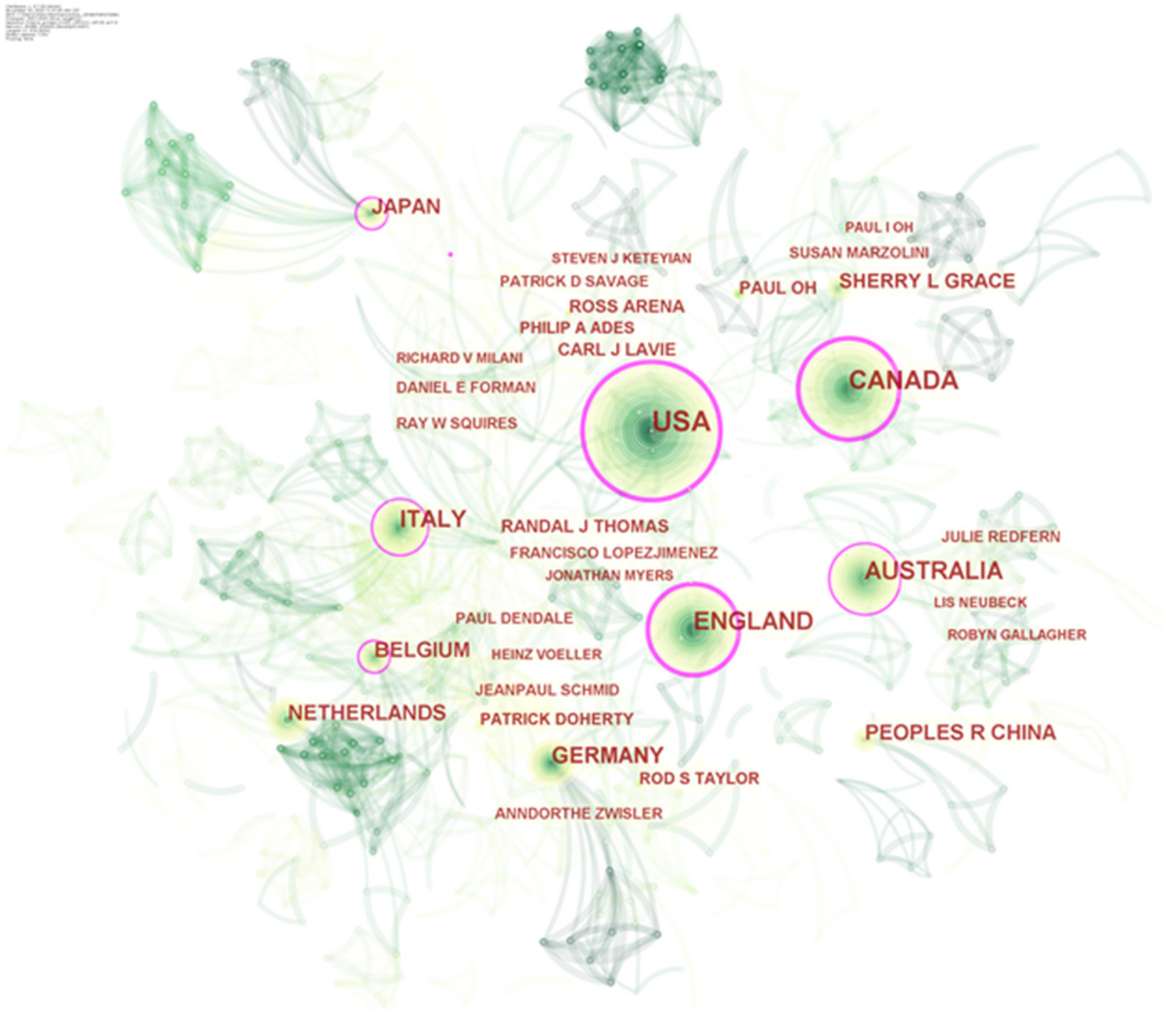
Authors–countries hybrid network of publications about cardiac rehabilitation (CR) and related research. *Circle node* represents output country or author of papers; *link between nodes* represents partnership.

Visualized knowledge mapping can provide information on influential research teams and potential collaborators and help researchers establish collaborative relationships (24). An analysis of the country distribution of these publications shows that the United States, Canada, and England are the most noticeable countries for co-authored papers, not only for the largest number of articles written in collaboration but also for the highest betweenness centrality (Table 1), revealing that these countries are highly outstanding in CR research, both quantitatively and qualitatively. Indeed, it was these countries that were among the first to focus on and implement CR, and several academic communities active in CR have been formed in these countries. Sherry L. Grace, full professor in the Faculty of Health at York University of Canada, is one of the representative figures. Professor Grace's research centers on optimizing post-acute cardiovascular care globally, as well as outcomes (including mental health). As the most prolific co-author who has made an important contribution to the field of CR (Table 1), she authored clinical practice guidelines internationally (25, 26) and led the development of the Canadian quality indicators for CR (27, 28), as well as policy positions on systematic referral and utilization (29). In the United States, Professor Ross Arena from the University of Illinois at Chicago is a moving force in the world of cardiovascular rehabilitation. He is devoted to exercise testing and training in patients diagnosed with cardiopulmonary dysfunction, as well as healthy living initiatives and policies that promote the healthspan (30–32). More importantly, he has conceived of and oversaw the successful implementation of several innovative healthy living initiatives in the academic, clinical, and community settings (33). We can see that, however, there are far more links between the nodes representing authors than between the nodes representing countries in Figure 2, which means that the interaction and the corporation between countries in the field of CR are relatively poor, and the form of partnership is still dominated by corporation within academic teams in each country.

**Table 1:** Top 5 productive countries and top 10 productive authors of publications about cardiac rehabilitation (CR) and related research.

**Rank**	**Country/author**	**Publication frequency**	**Burst**	**BC**
1	USA	1,529	8.51	0.24
2	Canada	747	–	0.24
3	England	588	5.96	0.22
4	Australia	532	–	0.17
5	Italy	385	–	0.18
1	Sherry L. Grace	158	–	0.05
2	Paul Oh	80	6.05	0.01
3	Ross Arena	69	5.03	0.02
4	Randal J. Thomas	66	4.45	0.05
5	Carl J. Lavie	65	3.36	0.02
6	Patrick Doherty	62	8.27	0.02
7	Rod S. Taylor	56	3.67	0.05
8	Philip A. Ades	56	6.14	0.01
9	Susan Marzolini	46	–	0
10	Anndorthe Zwisler	46	4.31	0.02

*BC, betweenness centrality*.

### Subject Categories Co-occurrence Analysis

**Figure 3:**
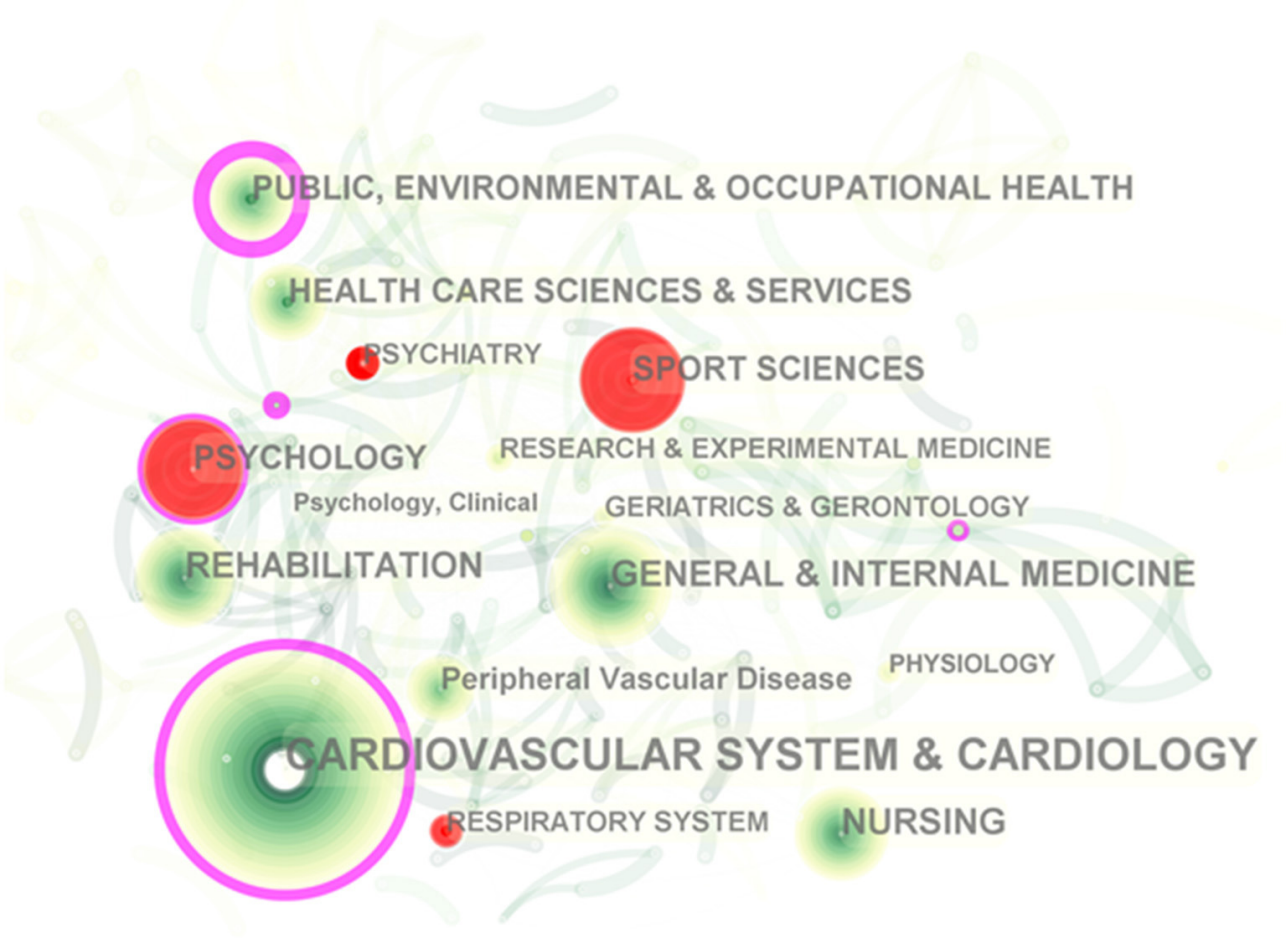
Subject category co-occurrence of publications about cardiac rehabilitation (CR) and related research. *Circle node* represents subject category; *link between nodes* represents interdisciplinary interaction of the literature.

The subject category (SC) in the Web of Science is based on journal–journal citation patterns and the judgment of domain experts, and then it is assigned to periodical, and finally, the papers included in the journal will be classified into the SC of the journal. Although this classification is controversial (34), we still use the subject co-occurrence function of CiteSpace to analyze the fields as SC in the literature due to its wide application and easy access (35), which contributes to constructing a correlation network of CR that demonstrates the interconnectedness of the disciplines and identifying high-impact disciplinary categories at the meso level. CR, a typical interdiscipline, is a comprehensive project for patients with CVDs (36), which is corroborated by disciplinary co-occurrence analysis. The results show varying degrees of interrelatedness between CR and lots of disciplines, and the involvement and utilization are the highest in clinical medicine, followed by public health management. Interestingly, CR also shows a strong crossover with sports science, a non-medical discipline (Figure 3 and Table 2). In Figure 3, the nodes denoting cardiology, public, environmental, and occupational health, and psychology are labeled with purple circles, namely with good betweenness centrality (BC > 0.1), suggesting that they are more impactful disciplines. The red nodes with positive burst detection are popular subjects for a certain period of time. For example, exercise prescription is a key component of CR, but clinicians experience difficulties in how to optimally prescribe exercise for patients with different cardiovascular disease risk factors (37), which requires sports medicine specialists to get involved in order to develop a personalized exercise program tailored to the needs of CVD patients. People with CVD are often accompanied by mental disorders, such as stress, anxiety, and depression, and it is a major challenge for heart rehabilitation (38–40). For such patients, the intervention of a professional psychologist or psychiatrist is urgently needed. In a word, CR has a comprehensive goal to aim for the optimal recovery of CVD patients physically, mentally, and socially. An ideal CR therapy demands multidisciplinary cooperation and integrative intervention, including medicine, exercise, nutrition, education, and psychological and social support, thus posing a huge challenge to interdisciplinary collaboration around CR. To obtain better development and application of CR, what need to be strengthened are breaking down the barriers between disciplines as well as accelerating the integration of transdisciplinary intelligence.

**Table 2:** Top 15 subject categories of publications about cardiac rehabilitation (CR) and related research.

**Rank**	**Subject category**	**Frequency**	**Burst**	**BC**
1	Cardiovascular system and cardiology	2,611	–	0.21
2	General and internal medicine	562	–	0.1
3	Nursing	516	–	0.07
4	Rehabilitation	490	–	0.07
5	Sport sciences	331	4.58	0.05
6	Public, environmental, and occupational health	330	–	0.44
7	Psychology	292	14.84	0.19
8	Health care sciences and services	248	–	0.06
9	Peripheral vascular disease	185	–	0.03
10	Geriatrics and gerontology	130	–	0.06
11	Psychiatry	130	8.34	0.02
12	Research and experimental medicine	116	6.91	0.08
13	Respiratory system	115	6.87	0.02
14	Physiology	84	–	0.1
15	Psychology, clinical	77	–	0.01

*BC, betweenness centrality*.

### Topic Distribution Analysis Based on Keywords

**Figure 4:**
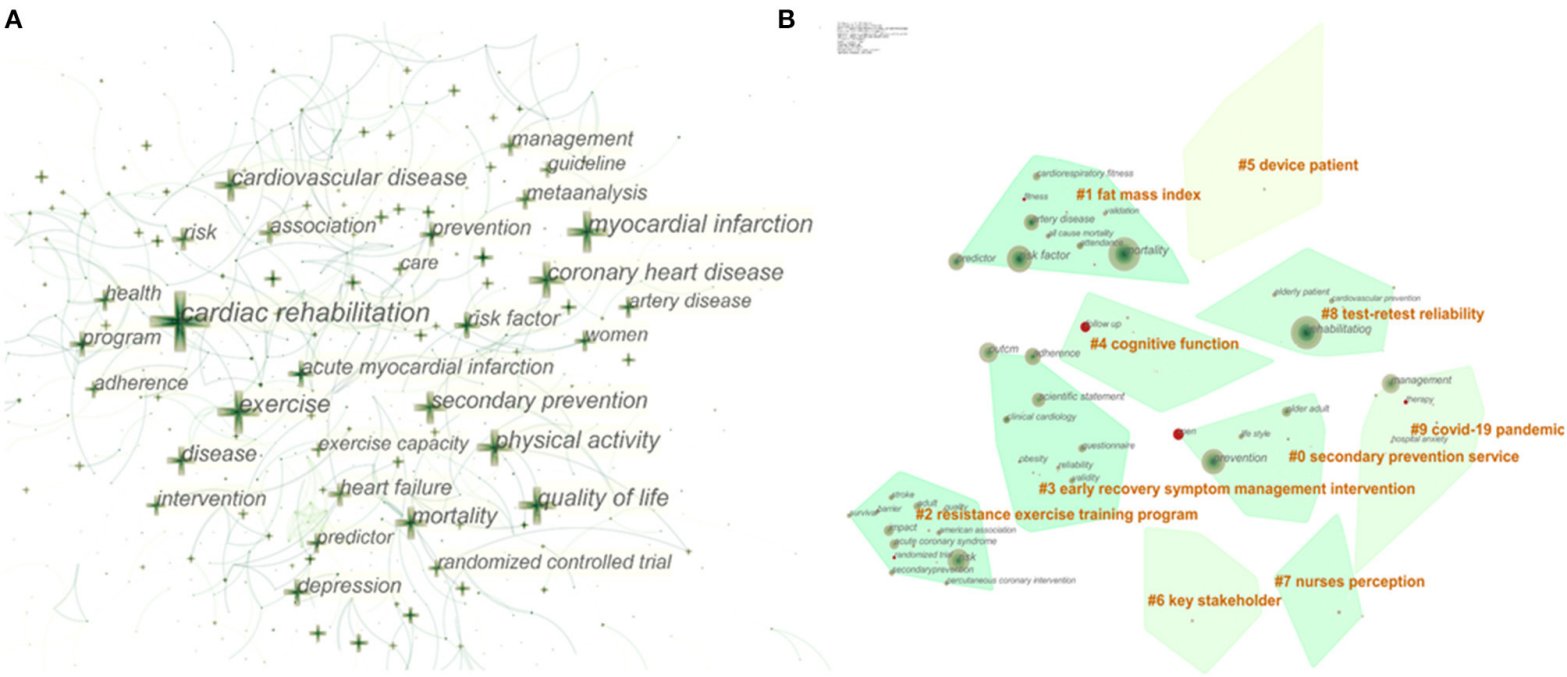
Keyword co-occurrence and clustering. **(A)** Network of the main keywords in publications about cardiac rehabilitation (CR) and related research. **(B)** Top 10 keyword clusters.

If subject co-occurrence is to explore research topics in a field at a meso level, keyword co-occurrence is to answer the specific questions in the subject from a micro perspective. As a highly condensed version of the paper's content, to some extent, keywords are able to summarize the theme of the paper simply and directly. The keyword co-occurrence network is a text content-based analytical approach. It sorts out the connections between the different topics in the discipline and helps readers get more acquainted with it through analyzing the co-occurring forms of keyword pairs in the same text. The co-occurrence results of the keywords in publications of CR and related research can be seen in Figure 4. The cross in Figure 4A indicates the keyword; the larger the size of the cross, the higher the frequency of the keyword. In fact, when the number of keywords is too large, it is difficult for us to generalize the research topics they belong to, and cluster analysis could assist with this problem. Figure 4B shows the top 10 keywords clustering based on the log-likelihood rate (LLR) algorithm. They encompass plenty of concerns in the field of CR, comprising participant (#5device patient, #6stakeholder), implementer (#7Nurses perception), content (#1 Fat mass index, #2 Resistance exercise training program, and #4 Cognitive function), purpose (#0 Secondary prevention service), requirements (#8 Test–retest reliability), and indications of CR (#9 COVID-19 pandemic).

The primary item that must be mentioned is the coronavirus disease 2019 (COVID-19) pandemic (cluster #9), which continues to attract global eyes throughout 2020 and will last for some time. As the pandemic is spreading all over the world, there are increasing reports of cardiac system damage and cardiovascular sequelae caused by COVID-19 (41, 42). Obviously, CR can play a significant role in the fight against this catastrophe, beneficial for patients with aggravation of preexisting CVD or secondary cardiovascular impairment due to COVID-19 (43). Secondly, most of the clusters involve the core components of CR, also known as prescription for CR, containing management of risk factors like overweight and obesity (cluster #1), psychosocial support (cluster #3), and exercise training (cluster #2), all of which are core modalities of CR recommended by international guidelines (36, 44). Last but not least, it is the evaluation of tests used for CR to ensure the reliability, stability, and consistency of the test results, such as the 6-min walk test, the 10-m incremental shuttle walk test, and other common outcome measurements in CR, which is crucial for the long-term validity of the tests for CR (45, 46). At the same time, screening out tests with high retest reliability makes it possible to compare and evaluate the efficacy in large clinical populations of CVD after recovery in the future. In brief, clustering based on keywords reveals the hot issues of CR microcosmically and helps us have a better sense of the topic distribution in the field of CR.

### Reference Co-citation Analysis

**Figure 5:**
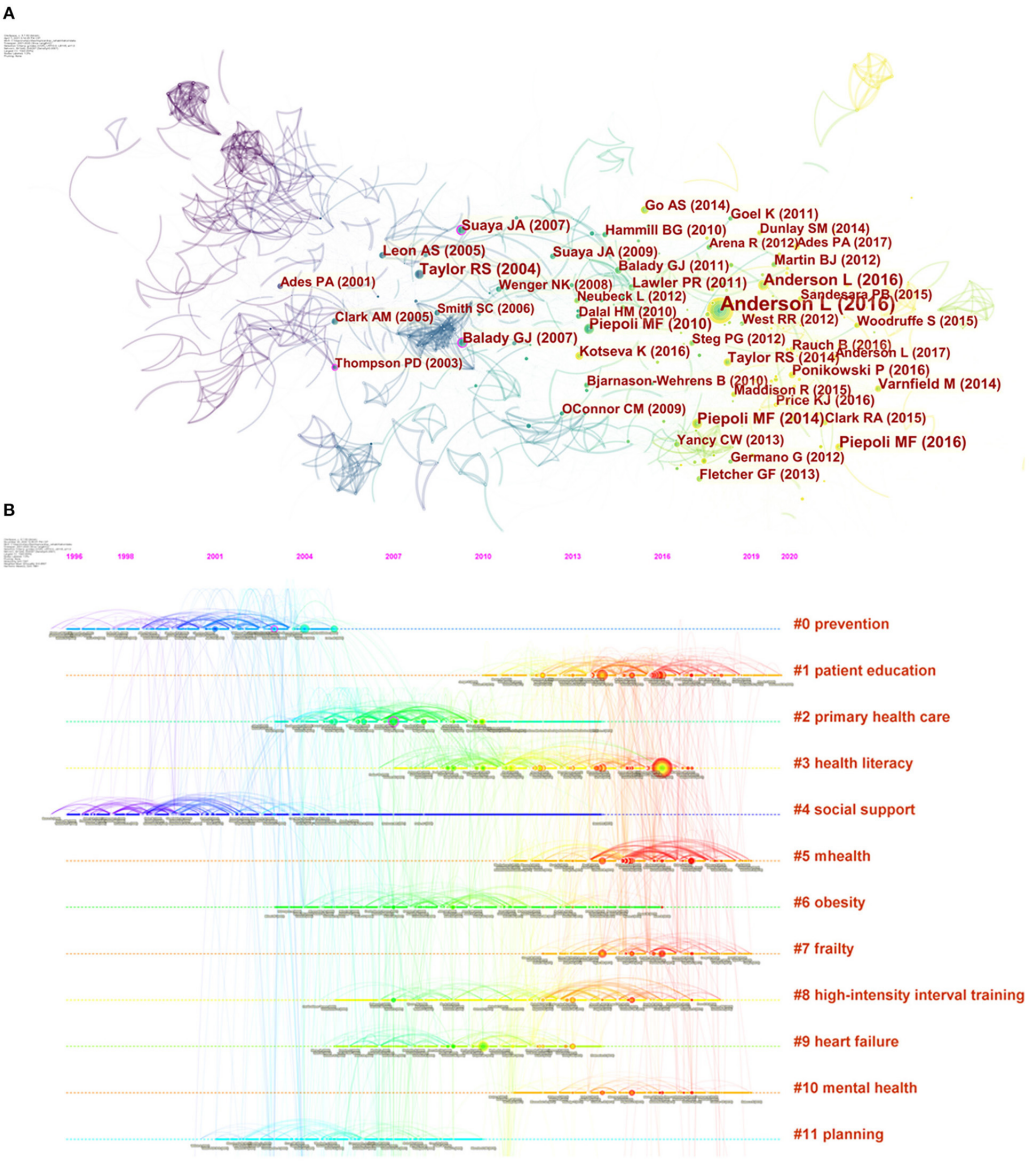
Co-citation network and timeline view of references cited by publications about cardiac rehabilitation (CR) and related research. **(A)** References co-citation network. *Circle node* represents reference; the *line between the nodes* indicates the frequency of the two references being cited at the same time. The betweenness centrality of the nodes with *purple circle* is >0.1. **(B)** Timeline view of references. Each *horizontal line* represents a cluster; the *circular nodes on the line* represent the top three most cited references in this time slice. The timeline is shown at the *top of the figure*, and the year corresponding to the node is its publication time. *Link between nodes* represents the co-citation relationship.

Co-citation analysis was put forward by Small and Marshakova in 1973 and later introduced into the analysis of co-citation of references, a phenomenon of two or more references being cited in the same literature (47, 48). By analyzing the clusters and pivotal nodes in the co-citation network, the knowledge structure of a research area and its changes can be uncovered. Figure 5 shows the co-citation network and timeline view of references on CR and related studies. The circular node in Figure 5A represents the reference, and the larger the size of the node, the higher is the citation frequency of the reference. Obviously, there are three nodes with a purple circle in the network; that is, the BC of the nodes is >0.1, which indicates that they are the critical turning points driving the development of CR. The first turning point occurred in 2003. A guideline jointly released by two subcommittees of AHA provides a clear definition of physical activity and exercise as well as their dose and intensity, and a comprehensive summary of prospective epidemiologic studies showing that physical activity could reduce the incidence of CAD events over the past half century (49). Therefore, it can provide clinicians with direct and detailed guidance when prescribing exercise to patients with atherosclerotic cardiovascular disease (ACD). This guideline broke the dilemma of having no guideline for prescribing exercise for adults with ACD and greatly improved the status of exercise in the prevention and treatment of CVD and the management of its risk factors, laying a solid evidence-based medical foundation for later recommending exercise as one of the core modalities of CR. The next two turning points both came in 2007. The former, again a guideline, was jointly released by the AHA and the American Association of Cardiovascular and Pulmonary Rehabilitation (AACPR). This update presented the latest information on the evaluation, interventions, and expected outcomes in each of the core components of CR/secondary prevention programs at that time, including baseline patient assessment, nutritional counseling, risk factor management, psychosocial interventions, and physical activity counseling and exercise training (36). Its content is more abundant and systematic than that of the guideline in 2003, providing more comprehensive guidance for the application of CR. The latter is a study that uses Medicare claims to evaluate national use patterns and predictors of CR use. The results directly reveal the underutilization and regional imbalance of CR in the United States, even though it is one of the countries with the most advanced CR referral system (50). To some extent, this survey served as a warning to healthcare policy makers that accelerating the widespread and balanced development of CR is imperative. In a word, these three crucial turnarounds could be considered as landmark events in the field of CR and have a profound impact on it.

CiteSpace also visualizes the research frontiers, knowledge base and their time span, as well as the literature that has played a key role in the evolution, in a unique way with a timeline view. The timeline view is a visualization method that combines the clustering and time slicing techniques. Items are ranked according to their early or late appearance after clustering, which both exhibit the topic distribution in the field and depict the trends and interconnections of the research topics over time. In Figure 5B, the cool-toned nodes (blue and green ones) represent earlier literature and the warm-toned nodes (yellow and red ones) represent more recent literature, with the earliest one dating back to 1996. A straight line at the same horizontal position indicates the set of all references belonging to the cluster, and the label of the cluster is located at the rightmost end of the line. The integrated network is divided into co-citation clusters of references. Citers to these references are considered as the research fronts associated with these clusters. Each cluster represents the knowledge fundamental of the underlying specialty. As can be seen in Figure 5B, in the early stage, CR-related research was dominated by exploration of its cardiovascular prevention and the importance of social support (cluster #0 and cluster #4). As strong evidence accumulated, these two questions were largely answered (51–53), so the research focus of CR had shifted to other aspects.

Despite overwhelming evidence that CR reduces risks and improves the quality of life and prognosis of CVD patients, the uptake and adherence to such programs among CVD patients remain below the recommended levels (54). Naturally, primary health care, planned CR intervention, and so on were immediately on the agenda (cluster #2 and cluster #11), and their implementation contributed to promoting patient participation and compliance in CR. Meanwhile, completing the prescription content of CR, such as the management of obesity and other risk factors (cluster #6), and tailoring rehabilitation programs for patients with heart failure were also the hot spots in this period (cluster #9). As stated in international guidelines and expert consensus, education, exercise training, and psychological support are three core modalities of CR (55). These core components are the current hot topics (cluster #1, cluster #3, cluster #4, cluster #8, and cluster #10), which are of great significance to improve CR prescription and promote the effective implementation of CR in the long run in this field. In addition, with advances in Internet technology and ubiquity of smart mobile devices, the availability of mobile health (mHealth) application that provides medical information and services via mobile devices such as pads and smart phones for CVD patients has markedly increased in recent years. There is no doubt that the elderly or mobility-impaired patients with CVD have the potential to benefit considerably from interventions that utilize mHealth (cluster #5 and #7). mHealth is extremely likely to be a complement or even an alternative to the traditional facility-based CR that is underutilized, and without much concern, there is also evidence that older patients may be willing to adopt these trendy technologies (56, 57).

In other aspects, the identification of core literature in a certain field generally depends on the frequency of citations, and references with the highest cited frequency, namely high-impact literature, are usually the main focus of the researchers. Table 3 shows the top 15 cited references with the highest frequency; their approximate content can be inferred from the titles. It is not difficult to find that the content of these most cited references is almost all centered on the clinical application of CR, and their types are mainly systematic review and meta-analysis and practice guideline or scientific statement. These high-quality systematic reviews provide important data and scientific evidence for the use of CR, especially exercise-based CR, in the CVD population, and they confirm that exercise-based CR lowers cardiovascular risk and mortality (6–8) and decreases reinfarction after myocardial infarction and heart failure-related hospital admissions (9, 10). Moreover, practice guidelines and scientific statements drafted by experts also provide practical advice and detailed guidance for the safe and effective application of CR and its effectiveness evaluation (13, 14, 36, 58–60). Clinical trial is one of the most important approaches of medical research, such as randomized controlled trial and cross-sectional study, assessing the participation, adherence, completion, and the effectiveness of CR in order to offer useful information and guidance for the long-term effective application of CR in the future (61, 62). In short, the co-citation analysis of CR-related references provides us with rich and valuable information to learn more about the evolution of the knowledge structure and changes of research hot spots in CR and helps to discover the core topic and key focus in this area.

**Table 3:** Top 15 most cited references of publications about cardiac rehabilitation (CR) and related research.

**Rank**	**Article title**	**Year**	**Total cited frequency**	**Average per year**
1	Exercise-Based Cardiac Rehabilitation for Coronary Heart Disease	2016	675	168.75
2	Exercise-Based Cardiac Rehabilitation for Coronary Heart Disease: Cochrane Systematic Review and Meta-analysis	2016	172	43.00
3	Exercise-Based Rehabilitation for Patients with Coronary Heart Disease: Systematic Review and Meta-analysis of Randomized Controlled Trials	2004	149	9.31
4	Secondary Prevention in the Clinical Management of Patients with Cardiovascular Diseases. Core Components, Standards and Outcome Measures for Referral and Delivery: A Policy Statement from the Cardiac Rehabilitation Section of the European Association for Cardiovascular Prevention and Rehabilitation. Endorsed by the Committee for Practice Guidelines of the European Society of Cardiology	2014	148	24.67
5	2016 European Guidelines on Cardiovascular Disease Prevention in Clinical Practice: The Sixth Joint Task Force of the European Society of Cardiology and Other Societies on Cardiovascular Disease Prevention in Clinical Practice (constituted by representatives of 10 societies and by invited experts) Developed with the special contribution of the European Association for Cardiovascular Prevention and Rehabilitation (EACPR)	2016	140	35.00
6	Secondary Prevention through Cardiac Rehabilitation: From Knowledge to Implementation. A position paper from the Cardiac Rehabilitation Section of the European Association of Cardiovascular Prevention and Rehabilitation	2010	124	12.40
7	Smartphone-Based Home Care Model Improved Use of Cardiac Rehabilitation in Postmyocardial Infarction Patients: Results from a Randomized Controlled Trial	2014	103	17.17
8	2016 ESC Guidelines for the Diagnosis and Treatment of Acute and Chronic Heart Failure: The Task Force for the Diagnosis and Treatment of Acute and Chronic Heart Failure of the European Society of Cardiology (ESC) Developed with the special contribution of the Heart Failure Association (HFA) of the ESC	2016	103	25.75
9	Cardiac Rehabilitation and Secondary Prevention of Coronary Heart Disease: An American Heart Association Scientific Statement from the Council on Clinical Cardiology (Subcommittee on Exercise, Cardiac Rehabilitation, and Prevention) and the Council on Nutrition, Physical Activity, and Metabolism (Subcommittee on Physical Activity), in collaboration with the American Association of Cardiovascular and Pulmonary Rehabilitation	2005	99	6.60
10	Use of Cardiac Rehabilitation by Medicare Beneficiaries after Myocardial Infarction or Coronary Bypass Surgery	2007	99	7.62
11	Core Components of Cardiac Rehabilitation/Secondary Prevention Programs: 2007 Update: A Scientific Statement from the American Heart Association Exercise, Cardiac Rehabilitation, and Prevention Committee, the Council on Clinical Cardiology; the Councils on Cardiovascular Nursing, Epidemiology and Prevention, and Nutrition, Physical Activity, and Metabolism; and the American Association of Cardiovascular and Pulmonary Rehabilitation	2007	97	7.46
12	Exercise Based Rehabilitation for Heart Failure	2014	96	16.00
13	Efficacy of Exercise-Based Cardiac Rehabilitation Post-myocardial Infarction: A Systematic Review and Meta-analysis of Randomized Controlled Trials	2011	94	10.44
14	EUROASPIRE IV: A European Society of Cardiology Survey on the Lifestyle, Risk Factor and Therapeutic Management of Coronary Patients from 24 European Countries	2016	86	21.50
15	Heart Disease and Stroke Statistics−2014 Update: A Report from the American Heart Association	2014	82	13.67

## Conclusions

With the accelerated aging of the global population and the dramatic increase in the number of patients surviving with CVD, how to reduce the risk of relapse and improve the prognosis of CVD patients is a major test for health care systems in all countries. CR is a shift from the traditional disease-centered model to a patient-centered biopsychosocial model, and its emergence is of great significance to patients with CVD. The visual analytic tool we utilized in this review plays an active role in supplementing traditional review and survey articles, and they are valuable in finding critical developments in the vast number of published studies.

In this review, our visual analysis of the literature on CR and related research over the last two decades has shown that CR has generally had a steady development during this period, but its development is quite unbalanced between areas and countries. Only a few developed Western countries such as Canada, the United States, and the UK have developed systematic and standardized CR models, as well as mature and well-established CR referral systems; awareness of and participation in CR are both insufficient in the vast majority of developing countries and regions. Correspondingly, several of the most active academic communities in CR have been formed in these countries, such as Professor Sherry Grace's team at York University in Canada and Professor Ross Arena's team at the University of Illinois in the United States. However, the interaction and collaboration around CR are poor among these leading countries and academic communities. In order for a balanced and widespread development of CR globally, it is urgent to strengthen transnational and cross-team cooperation, which requires the involvement of more international organizations specialized in CR. In addition, CR has a typical interdisciplinary characteristic, and its long-term and healthy development cannot be achieved without the coordinated development and mutual integration of clinical medicine, basic medicine, psychology, public health management, sports science, and even computer science. It means that more attention should be paid to the cultivation of compound talents with medical background in future medical education.

The keyword clustering analysis of the original documents and the co-citation analysis of the references reflect the trends and hot spots of CR. Early CR-related studies mainly focused on its cardiovascular prevention and social support, followed by primary health care and planned CR interventions to refine the social support for CR at the institutional and methodological levels. Then came the focus on specific populations, such as patients with obesity or heart failure, emphasizing the individualization of CR programs. The current hot spots are the three core modalities of CR, namely education, exercise training, and psychological support, particularly centering at exercise prescription for CR, including weight management, resistance exercise training program, and high-intensity interval training. At the same time, mHealth, born from the wave of Internet popularity, has started to emerge and become popular. There is no doubt that this trend of “Internet plus healthcare” will continue to prevail. Besides, it must be mentioned that CR is likely to play a critical role in improving cardiovascular damage and sequelae caused by COVID-19 as it continues to spread worldwide. Finally, there were three pivotal turning points that had driven the development of CR. The first was the guideline on physical activity and exercise in patients with ACD published by the AHA in 2003, followed by the systematic guideline on CR jointly issued by AHA and AACPR in 2007, and the last one is a study on the prediction of CR utilization. They have gradually contributed to the development of CR.

In conclusion, this work has provided some useful information for obtaining knowledge about CR as a long-established and innovative interdiscipline, identified potential collaborators for researchers interested in CR, and discovered research trends and hot topics. At the same time, we expect that our work will help more medical practitioners and patients with CVD, especially those in developing countries and regions where heart rehabilitation still lags behind. A deeper understanding of CR can inspire people's interest in it, which will be beneficial to the treatment and prognosis of the hundreds of millions of populations suffering from CVD worldwide.

## Author Contributions

YHH, GZY, and JJS devised the research plan and established the methodology. GZY, JJS, QLJ, SQS, and YHH wrote the original draft. XPZ, YZ, and SS were in charge of software, literature retrieval, and visualization. YHH, GZY, and JJS modified and polished the manuscript. All authors contributed to the article and approved the submitted version.

## Conflict of Interest

The authors declare that the research was conducted in the absence of any commercial or financial relationships that could be construed as a potential conflict of interest.
